# Supplementary material for: Ceramic Scaffolds in a Vacuum Suction Handle for Intraoperative Stromal Cell Enrichment
Source: Int J Mol Sci. 2020 Sep 2;21(17):6393. doi: 10.3390/ijms21176393 (PMC7504718; doi:10.3390/ijms21176393)
Supplement: Supplementary file 1 [file ijms-21-06393-s001.zip › Supplementary File 1 - Ethical Vote.pdf]

MEDIZINISCHE FAKULTÄT
DER UNIVERSITÄT DUISBURG-ESSEN

Ethik-Kommission
Robert-Koch-Str. 9-11 45147 Essen
Fax: +49 (0) 201/723-5837
ethikkommission@uk-essen.de
www.uni-due.de/ethikkommission

Vorsitzende: Prof. Dr. U. Schara

Leiterin der Geschäftsstelle: Dr. S. Bein
sabine.bein@uk-essen.de

Sekretariat: M. Oniangue-Ndza
☎ +49 (0) 201 / 723-3637
monika.oniangue-ndza@uk-essen.de

Sekretariat: U. van Saarloos
☎ +49 (0) 201 / 723-1847
ulla.vansaarloos@uk-essen.de

Mitarbeiterin: E. Ankert
elisa.ankert@uk-essen.de

Mitarbeiterin: N.N.
N.N.@uk-essen.de

Mitarbeiterin: Dr. S. Gent
sabine.gent@uk-essen.de

Mitarbeiterin: Dr. A. Gossens
anabel.gossens@uk-essen.de

Ethik-Kommission Medizinische Fakultät der Universität Duisburg- Essen

Herrn
Dr. André Busch
Klinik für Orthopädie und Unfallchirurgie
-im Hause-

Essen, den 19. Februar 20 / EA

**Studientitel:**	**Chirurgisches Saugersystem mit integriertem keramischen Knochenersatzstoff In vitro Untersuchung der Kombination aus Blut-/Gewebekomposit und ß-Tricalciumphosphatkeramik**
**Antragsteller:**	**Dr. Busch, Zentrum für Orthopädie und Unfallchirurgie**
**Unser Zeichen:**	**19-8822-BO**

Sehr geehrter Herr Dr. Busch,

als Vorsitzende habe ich im Auftrag der Mitglieder der Ethik-Kommission der Medizinischen Fakultät der Universität Duisburg-Essen Ihren o. g. Antrag geprüft.

Auf der Grundlage der übersandten Unterlagen und dem derzeitigen Informationsstand sieht die Ethik-Kommission keinen Anlass, ethische oder rechtliche Einwände gegen diese Studie zu erheben.

Die Kommission bittet jedoch die folgenden Hinweise zu beachten:

<u>Hinweise zur Patienteninformation und Einwilligungserklärung:</u>

1. Freiwilligkeit der Teilnahme: Bitte ergänzen Sie in der Informationsschrift wie bei Widerruf mit den Proben und Daten der Patienten verfahren wird.
2. Freiwilligkeit der Teilnahme: Bitte ergänzen Sie, dass ein Widerruf schriftlich und mündlich möglich ist.
3. Bitte geben Sie zusätzlich in der Einwilligungserklärung an wie lange die erhobenen Daten und Proben gespeichert werden.
4. Bitte reichen Sie die Zustimmung des hiesigen Datenschutzbeauftragten nach. Bei der Zustimmung des Datenschutzbeauftragten handelt es sich um ein formales Dokument, das Sie

Universitätsklinikum Essen

jeder Zeit nachreichen können. Mit der Durchführung der klinischen Prüfung kann bereits vor Einreichung des Dokuments begonnen werden.

Bitte lassen Sie der Ethik-Kommission die entsprechend modifizierte Fassung der Patienteninformation und Einwilligungserklärung mit den eindeutig gekennzeichneten Änderungen und einer neuen Versionsnummer versehen in einfacher Ausfertigung zukommen.

Die Kommission geht davon aus, dass diese Studie gemäß den Prinzipien des Weltärztebundes, niedergelegt in der Deklaration von Helsinki, und den nationalen Vorschriften für die Durchführung klinischer Studien durchgeführt wird.

Die Verantwortung für die Studie und ihre Durchführung verbleibt uneingeschränkt bei den Prüfärzten und wird nicht von der Ethik-Kommission übernommen.

Datenschutzrechtliche Aspekte von Forschungsvorhaben werden durch die Ethik-Kommission grundsätzlich nur kursorisch geprüft. Diese zustimmende Bewertung ersetzt mithin nicht die Konsultation des zuständigen Datenschutzbeauftragten.

Bitte teilen Sie der Ethik-Kommission das Datum des Einschlusses des ersten Patienten in die Studie mit.

Die Kommission erwartet, dass sie über alle für die Patienten relevanten Änderungen des Prüfplans informiert wird. Nach Abschluss der Studie erbittet die Kommission einen kurzen Bericht über den Ausgang der Studie.

Die Kommission empfiehlt die Studie beim „Deutsches Register Klinischer Studien“ (www.drks.de) zu registrieren. Die Medizinische Fakultät der Universität Duisburg-Essen führt darüber hinaus ein Register für klinische Studien ihrer Mitglieder.

Die Kommission wünscht Ihnen und allen Studienteilnehmern viel Erfolg.

Mit freundlichen Grüßen

U. Schara

Prof. Dr. U. Schara
