# Supplementary material for: Ceramic Scaffolds in a Vacuum Suction Handle for Intraoperative Stromal Cell Enrichment
Source: Int J Mol Sci. 2020 Sep 2;21(17):6393. doi: 10.3390/ijms21176393 (PMC7504718; doi:10.3390/ijms21176393)
Supplement: Supplementary file 1 [file ijms-21-06393-s001.zip › Supplementary File 2 - Proteome Profiler Array Human XL Cytokine.pdf]

Proteome Profiler™ Array

Human XL Cytokine Array Kit

Catalog Number ARY022

For the parallel determination of the relative levels of selected human cytokines.

This package insert must be read in its entirety before using this product.
For research use only. Not for use in diagnostic procedures.

TABLE OF CONTENTS

SECTION	PAGE
INTRODUCTION	1
PRINCIPLE OF THE ASSAY	1
TECHNICAL HINTS	1
MATERIALS PROVIDED & STORAGE CONDITIONS	2
OTHER SUPPLIES REQUIRED	3
SUPPLIES REQUIRED FOR CELL LYSATE SAMPLES	3
SUPPLIES REQUIRED FOR TISSUE LYSATE SAMPLES	3
SAMPLE COLLECTION & STORAGE	4
REAGENT PREPARATION	5
PRECAUTIONS	5
ARRAY PROCEDURE	6
DATA ANALYSIS	8
PROFILING PROTEINS IN CELL CULTURE SUPERNATES	9
PROFILING PROTEINS IN CELL LYSATES	11
PROFILING PROTEINS IN TISSUE LYSATES	13
PROFILING PROTEINS IN PBMC SUPERNATES & BODY FLUIDS	14
APPENDIX	16

MANUFACTURED AND DISTRIBUTED BY:

USA & Canada | R&D Systems, Inc.
614 McKinley Place NE, Minneapolis, MN 55413, USA
TEL: (800) 343-7475   (612) 379-2956   FAX: (612) 656-4400
E-MAIL: info@RnDSystems.com

DISTRIBUTED BY:

UK & Europe | R&D Systems Europe, Ltd.
19 Barton Lane, Abingdon Science Park, Abingdon OX14 3NB, UK
TEL: +44 (0)1235 529449   FAX: +44 (0)1235 533420
E-MAIL: info@RnDSystems.co.uk

China | R&D Systems China Co., Ltd.
24A1 Hua Min Empire Plaza, 726 West Yan An Road, Shanghai PRC 200050
TEL: +86 (21) 52380373   FAX: +86 (21) 52371001
E-MAIL: info@RnDSystemsChina.com.cn

INTRODUCTION

Cytokines, chemokines and growth factors are extracellular signaling molecules that mediate cell to cell communication. These molecules are released from cells and have critical roles in many biological processes such as cellular growth, differentiation, gene expression, migration, immunity and inflammation. In most biological processes, multiple cytokines operate in a large network, where the action of one cytokine is regulated by the presence or absence of other cytokines. The Human XL Cytokine Array Kit is a rapid, sensitive, and economic tool to simultaneously detect cytokine differences between samples. The relative expression levels of 102 human soluble proteins can be determined without performing numerous immunoassays.

PRINCIPLE OF THE ASSAY

Capture and control antibodies have been spotted in duplicate on nitrocellulose membranes. Cell culture supernates, cell lysates, serum, plasma, human milk, urine, saliva, or tissue lysates are diluted and incubated overnight with the Proteome Profiler Human XL Cytokine Array. The membrane is washed to remove unbound material followed by incubation with a cocktail of biotinylated detection antibodies. Streptavidin-HRP and chemiluminescent detection reagents are then applied, and a signal is produced at each capture spot corresponding to the amount of protein bound. Refer to the Appendix for a list and coordinates of analytes and controls.

TECHNICAL HINTS

- FOR RESEARCH USE ONLY. NOT FOR USE IN DIAGNOSTIC PROCEDURES.
- This kit should not be used beyond the expiration date on the kit label.
- **Do not mix or substitute reagents with those from other lots or sources. Substitution of some high intensity chemiluminescent reagents for Chemi Reagents 1 and 2 may cause either increased background or diminished signal depending on the reagent.**
- Any variation in sample handling, buffers, operator, pipetting technique, washing technique, and incubation time or temperature can alter the performance of the kit.
- The array membranes are validated for single use only.
- Always use gloved hands and flat-tipped tweezers to handle the membranes.
- Pick up the membranes from the edge on the side with the identification number avoiding the area with the printed antibodies.
- A thorough and consistent wash technique is essential for proper assay performance. Individual arrays should be washed in separate containers to minimize background. Wash Buffer should be removed completely from the membrane before proceeding to the next step.
- Do not allow the membrane to dry out. This will cause high background.
- Avoid microbial contamination of reagents and buffers.
- Other proteins present in biological samples do not necessarily interfere with the measurement of analytes in samples. Until these proteins have been tested with the Proteome Profiler Array kit, the possibility of interference cannot be excluded.
- For a procedure demonstration video, please visit: www.RnDSystems.com/ProteomeProfilerVideo.

MATERIALS PROVIDED & STORAGE CONDITIONS

Store the unopened kit at 2-8 °C. Do not use past kit expiration date.

PART	PART #	DESCRIPTION	STORAGE OF OPENED/ RECONSTITUTED MATERIAL
**Human XL Cytokine Array**	894660	4 nitrocellulose membranes each containing 102 different capture antibodies printed in duplicate.	Return unused membranes to the foil pouch containing the desiccant pack. Reseal along entire edge of the zip-seal. May be stored for up to 3 months at 2-8 °C.*
**Array Buffer 4**	895022	21 mL of a buffered protein base with preservatives. *May contain a precipitate. Mix well before and during use.*	May be stored for up to 3 months at 2-8 °C.*
**Array Buffer 6**	893573	2 vials (21 mL/vial) of a buffered protein base with preservatives.	
**Wash Buffer Concentrate**	895003	2 vials (21 mL/vial) of a 25-fold concentrated solution of buffered surfactant with preservative. *May turn yellow over time.*	
**Detection Antibody Cocktail, Human XL Cytokine Array**	894661	1 vial of biotinylated antibody cocktail; lyophilized.	
**Streptavidin-HRP**	893019	200 µL of streptavidin conjugated to horseradish-peroxidase.	
**Chemi Reagent 1**	894287	2.5 mL of stabilized hydrogen peroxide with preservative.	
**Chemi Reagent 2**	894288	2.5 mL of stabilized luminol with preservative.	
**4-Well Multi-dish**	607544	Clear 4-well rectangular multi-dish.	Store at room temperature.
**Transparency Overlay Template**	607156	1 transparency overlay template for coordinate reference.	

* Provided this is within the expiration date of the kit.

For research use only. Not for use in diagnostic procedures.

OTHER SUPPLIES REQUIRED

- Pipettes and pipette tips
- Gloves
- Deionized or distilled water
- Rocking platform shaker
- Microcentrifuge
- A plastic container with the capacity to hold 50 mL (for washing the arrays)
- Plastic transparent sheet protector (trimmed to 10 cm x 12 cm and open on three sides)
- Plastic wrap
- Paper towels
- Absorbent lab wipes (KimWipes® or equivalent)
- Autoradiography cassette
- Film developer
- X-ray film (Kodak® BioMax™ Light-1, Catalog # 1788207) or equivalent
- Flat-tipped tweezers
- Flatbed scanner with transparency adapter capable of transmission mode
- Computer capable of running image analysis software and Microsoft® Excel

SUPPLIES REQUIRED FOR CELL LYSATE SAMPLES

- Phosphate-Buffered Saline (PBS)
- Lysis Buffer 17 (R&D Systems, Catalog # 895943)
- Aprotinin (Sigma, Catalog # A6279)
- Leupeptin (Tocris, Catalog # 1167)
- Pepstatin (Tocris, Catalog # 1190)

SUPPLIES REQUIRED FOR TISSUE LYSATE SAMPLES

- Protease Inhibitor Cocktail (Sigma, Catalog # P8340)
- Igepal® CA-630 (Sigma, Catalog # I3021)
- Sodium deoxycholate (Sigma, Catalog # D6750)
- Sodium dodecyl sulfate (Sigma, Catalog # L6026)

SAMPLE COLLECTION & STORAGE

The sample collection and storage conditions listed below are intended as general guidelines. Sample stability has not been evaluated.

Since the Human XL Cytokine Array detects relative expression levels of individual analytes, it is important to include appropriate control samples.

Note: *Sample amount may be empirically adjusted to attain optimal sensitivity with minimal background. Suggested starting ranges are: 200-500 µL for cell culture supernates, 100-200 µg for cell and tissue lysates, and 50-200 µL for serum, plasma, human milk, urine, and saliva samples.*

Cell Culture Supernates - Remove particulates by centrifugation. Assay immediately or aliquot and store samples at ≤ -20 °C. Avoid repeated freeze-thaw cycles.

Cell Lysates - Rinse cells with PBS, making sure to remove any remaining PBS before adding Lysis Buffer 17 supplemented with 10 µg/mL Aprotinin, 10 µg/mL Leupeptin, and 10 µg/mL Pepstatin. Solubilize cells at 1 x 10^7 cells/mL in this buffer. Pipette up and down to resuspend and rock the lysates gently at 2-8 °C for 30 minutes. Microcentrifuge at 14,000 x g for 5 minutes, and transfer the supernate into a clean test tube. Quantitation of sample protein concentration using a total protein assay is recommended. Assay immediately or aliquot and store at ≤ -70 °C. Avoid repeated freeze-thaw cycles.

Serum - Allow blood samples to clot for 30 minutes at room temperature before centrifuging for 15 minutes at 1000 x g. Remove serum and assay immediately or aliquot and store samples at ≤ -20 °C. Avoid repeated freeze-thaw cycles.

Plasma - Collect plasma using EDTA or heparin as an anticoagulant. Centrifuge for 15 minutes at approximately 1000 x g within 30 minutes of collection. Assay immediately or aliquot and store samples at ≤ -20 °C. Avoid repeated freeze-thaw cycles.

Human Milk - Centrifuge for 15 minutes at 1000 x g at 2-8 °C. Collect the aqueous fraction and repeat this process a total of 3 times. Assay immediately or aliquot and store samples at ≤ -20 °C. Avoid repeated freeze-thaw cycles.

Urine - Collect urine and centrifuge to remove particulate matter. Assay immediately or aliquot and store at ≤ -20 °C. Avoid repeated freeze-thaw cycles.

Saliva - Collect saliva in a tube and centrifuge for 5 minutes at 10,000 x g. Collect the aqueous layer, and assay immediately or aliquot and store samples at ≤ -20 °C. Avoid repeated freeze-thaw cycles.

Tissue Lysates - Excise tissue and place in Tissue Lysis buffer (0.5% Igepal, 0.5% sodium deoxycholate, 0.1% sodium dodecyl sulfate, 50 mM Tris-HCl (pH 7.5), and 150 mM NaCl) with Protease Inhibitor Cocktail. Homogenize tissue and centrifuge at 1000 x g for 10 minutes at 2-8 °C to remove cellular debris. Transfer supernate to a new tube. Quantitation of sample protein concentration using a total protein assay is recommended. Assay immediately or aliquot and store samples at ≤ -70 °C. Avoid repeated freeze-thaw cycles.

REAGENT PREPARATION

Bring all reagents to room temperature before use.

Note: *High levels of some proteins are found in saliva. It is recommended that a mask and gloves be used to protect kit reagents from contamination.*

Human XL Cytokine Array - Immediately before use, remove each membrane to be used from between the protective sheets with a flat-tipped tweezers. **Handle the membranes with gloved hands and flat-tipped tweezers only.**

Detection Antibody Cocktail - Before use, reconstitute the Human XL Cytokine Detection Antibody Cocktail in 200 µL of deionized or distilled water.

1X Array Buffer 4/6 - *Array Buffer 4 may contain a precipitate. Mix well before and during use.* Add 4 mL of Array Buffer 4 to 8 mL of Array Buffer 6. Prepare fresh for each use.

1X Wash Buffer - If crystals have formed in the concentrate, warm the bottles to room temperature and mix gently until the crystals have completely dissolved. Add 40 mL of Wash Buffer Concentrate to 960 mL of deionized or distilled water.

Chemi Reagent Mix - Chemi Reagents 1 and 2 should be mixed in equal volumes within 15 minutes of use. **Protect from light. 1 mL of the resultant mixture is required per membrane.**

1X Streptavidin-HRP - Immediately before use, dilute the Streptavidin-HRP in Array Buffer 6. See vial label for dilution factor.

PRECAUTIONS

Chemi Reagents 1 and 2 contain Boric Acid which is suspected of damaging fertility or the unborn child.

High levels of some proteins are found in saliva. It is recommended that a mask and gloves be used to protect kit reagents from contamination.

Some components in this kit contain ProClin® which may cause an allergic skin reaction. Avoid breathing mist.

Wear protective gloves, clothing, eye, and face protection. Wash hands thoroughly after handling. Please refer to the MSDS on our website prior to use.

ARRAY PROCEDURE

Bring all reagents to room temperature before use. Keep samples on ice. To avoid contamination, wear gloves while performing the procedures.

Note: *High levels of some proteins are found in saliva. It is recommended that a mask and gloves be used to protect kit reagents from contamination.*

1. Prepare all reagents and samples as directed in the previous sections.
2. Pipet 2.0 mL of Array Buffer 6 into each well of the 4-Well Multi-dish. Array Buffer 6 serves as a block buffer.
3. Place each membrane in a separate well. The number on the membrane should be facing upward.

   Note: *Upon contact with Array Buffer 6, the blue dye from the spots will disappear, but the capture antibodies are retained in their specific locations.*
4. Incubate for one hour on a rocking platform shaker. Orient the 4-Well Multi-dish so that each membrane rocks end to end in its well
5. While the arrays are blocking, prepare samples by diluting the desired quantity to a final volume of 1.5 mL with Array Buffer 6.
6. Aspirate Array Buffer 6 from the wells of the 4-Well Multi-dish and add the prepared samples. Place the lid on the 4-Well Multi-dish.
7. Incubate overnight at 2-8 °C on a rocking platform shaker.

   Note: *A shorter incubation time may be used if optimal sensitivity is not required.*
8. Carefully remove each membrane and place into individual plastic containers with 20 mL of 1X Wash Buffer. Rinse the 4-Well Multi-dish with deionized or distilled water and dry thoroughly.
9. Wash each membrane with 1X Wash Buffer for 10 minutes on a rocking platform shaker. Repeat two times for a total of three washes.
10. For each array, add 30 µL of Detection Antibody Cocktail to 1.5 mL of 1X Array Buffer 4/6. Pipette 1.5 mL per well of diluted Detection Antibody Cocktail into the 4-Well Multi-dish.
11. Carefully remove each array from its wash container. Allow excess Wash Buffer to drain from the array. Return the array to the 4-Well Multi-dish containing the diluted Detection Antibody Cocktail, and cover with the lid.
12. Incubate for 1 hour on a rocking platform shaker.
13. Wash each array as described in steps 8 and 9.

For research use only. Not for use in diagnostic procedures.

14. Pipette 2.0 mL of 1X Streptavidin-HRP into each well of the 4-Well Multi-dish.
15. Carefully remove each membrane from its wash container. Allow excess Wash Buffer to drain from the membrane. Return the membrane to the 4-Well Multi-dish containing the 1X Streptavidin-HRP. Cover the wells with the lid.
16. Incubate for 30 minutes at room temperature on a rocking platform shaker.
17. Wash each array as described in steps 8 and 9.

    Note: *Complete the remaining steps without interruption.*

18. Carefully remove each membrane from its wash container. Allow excess Wash Buffer to drain from the membrane by blotting the lower edge onto paper towels. Place each membrane on the bottom sheet of the plastic sheet protector with the identification number facing up.
19. Pipette 1.0 mL of the prepared Chemi Reagent Mix evenly onto each membrane.

    Note: *Using less than 1.0 mL of Chemi Reagent Mix per membrane may result in incomplete membrane coverage.*

20. Carefully cover with the top sheet of the plastic sheet protector. Gently smooth out any air bubbles and ensure Chemi Reagent Mix is spread evenly to all corners of each membrane. Incubate for 1 minute.
21. Position paper towels on the top and sides of the plastic sheet protector containing the membranes and carefully squeeze out excess Chemi Reagent Mix.
22. Leaving membranes on the bottom plastic sheet protector, cover the membranes with plastic wrap taking care to gently smooth out any air bubbles. Wrap the excess plastic wrap around the back of the sheet protector so that the membranes and sheet protector are completely wrapped.
23. Place the membranes with the identification numbers facing up in an autoradiography film cassette.

    Note: *Use an autoradiography cassette that is not used with radioactive isotope detection.*

24. Expose membranes to X-ray film for 1-10 minutes. Multiple exposure times are recommended.

DATA ANALYSIS

The positive signals seen on developed film can be quickly identified by placing the transparency overlay template on the array image and aligning it with the pairs of reference spots in three corners of each array. The stamped identification number on the array should be placed on the left hand side. The location of controls and capture antibodies is listed in the Appendix.

Note: *Reference spots are included to align the transparency overlay template and to demonstrate that the array has been incubated with Streptavidin-HRP during the assay procedure.*

Pixel densities on developed X-ray film can be collected and analyzed using a transmission-mode scanner and image analysis software.

1. Create a template to analyze pixel density in each spot of the array.
2. Export signal values to a spreadsheet file for manipulation in a program such as Microsoft Excel.
3. Determine the average signal (pixel density) of the pair of duplicate spots representing each analyte.
4. Subtract an averaged background signal from each spot. Use a signal from a clear area of the array or negative control spots as a background value.
5. Compare corresponding signals on different arrays to determine the relative change in analyte levels between samples.

Human XL Cytokine Array Coordinates

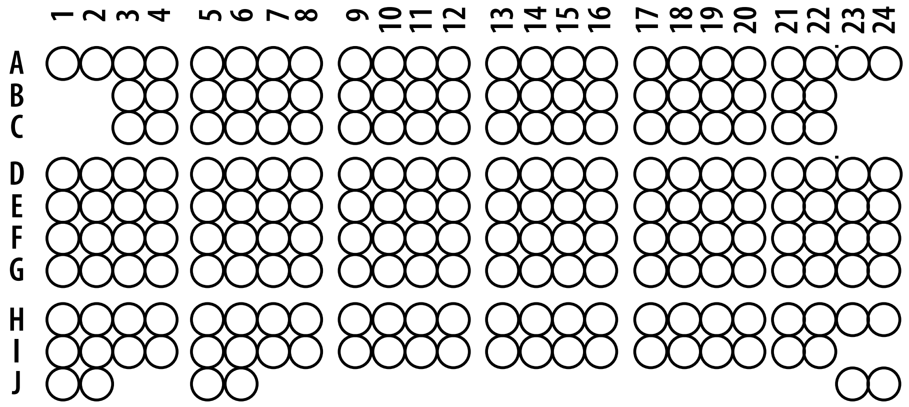

This image is not to scale. It is for coordinate reference only.
Please use the transparency overlay for analyte identification.

PROFILING PROTEINS IN CELL CULTURE SUPERNATES

The Human XL Cytokine Array detects multiple cytokines, chemokines, growth factors and other soluble proteins in cell culture supernates. Cells were untreated or treated as indicated below. 500 µL of cell culture supernate was run on each array. Data shown are from a 10 minute exposure to X-ray film. Profiles of mean spot pixel density were created using a transmission-mode scanner and image analysis software.

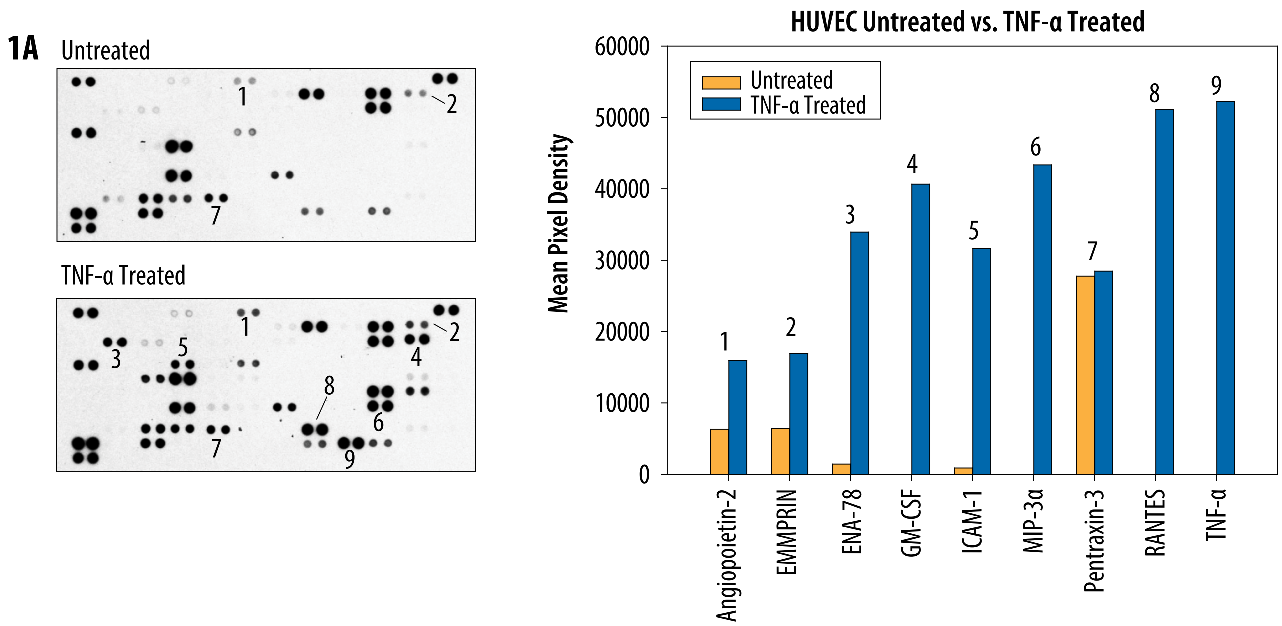

Figure 1A: HUVEC human umbilical vein endothelial cells were untreated or treated with 100 µg/mL recombinant human TNF-α (R&D Systems, Catalog # 210-TA) for 24 hours.

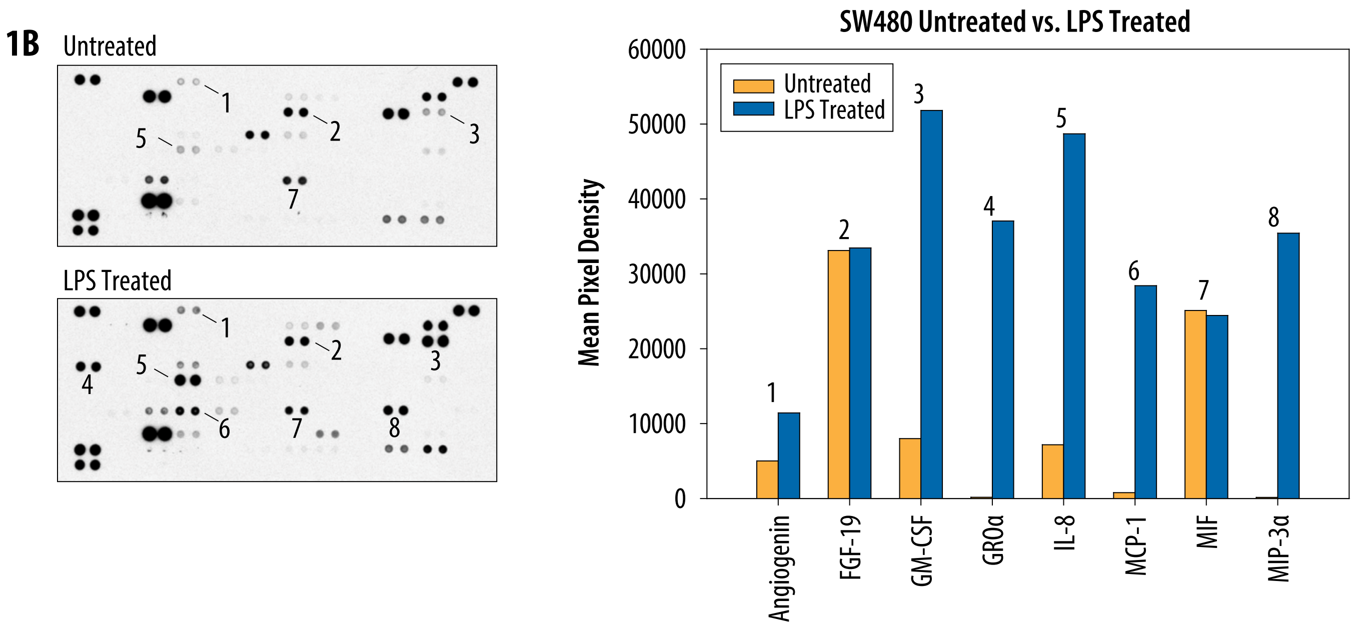

Figure 1B: SW480 human colorectal adenocarcinoma cells were untreated or treated with 100 ng/mL of LPS for 24 hours.

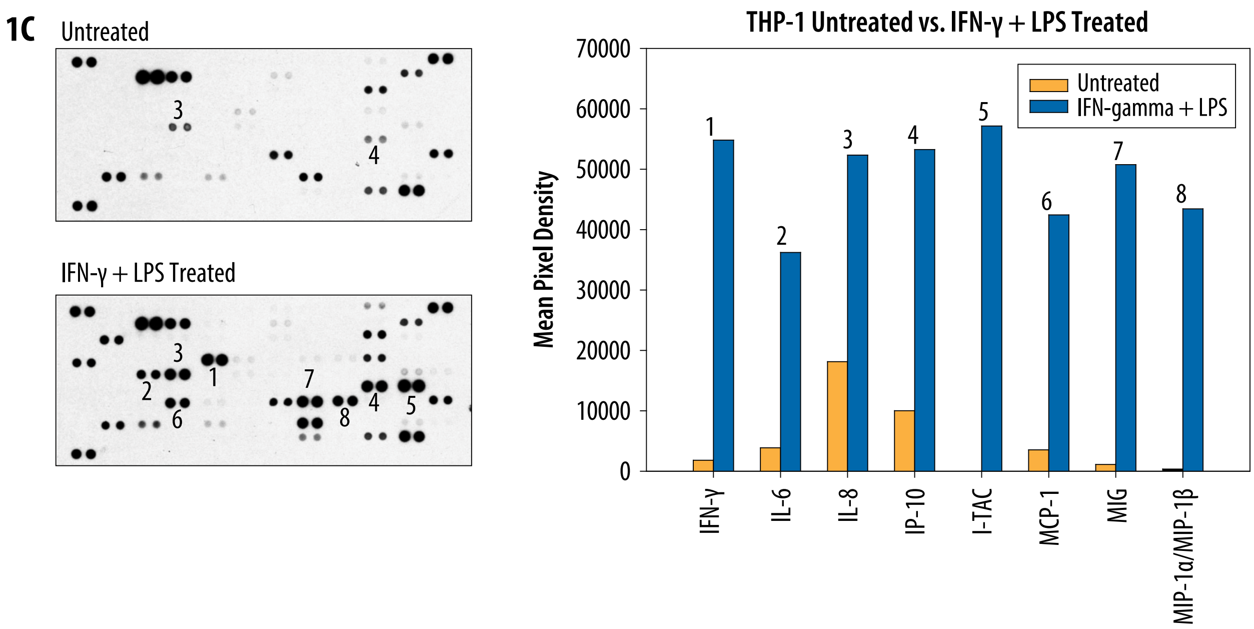

Figure 1C: THP-1 human acute monocytic leukemia cells were untreated or treated with 1 µg/mL of recombinant human IFN-γ (R&D Systems, Catalog # 285-IF) for 16 hours and 1 µg/mL of LPS for 8 hours.

For research use only. Not for use in diagnostic procedures.

PROFILING PROTEINS IN CELL LYSATES

The Human XL Cytokine Array detects multiple cytokines, chemokines, growth factors and other soluble proteins in cell lysates. Cells were either untreated or treated as indicated below. 200 µg of cell lysate was run on each array. Data shown are from a 5 minute exposure to X-ray film. Profiles of mean spot pixel density were created using a transmission-mode scanner and image analysis software.

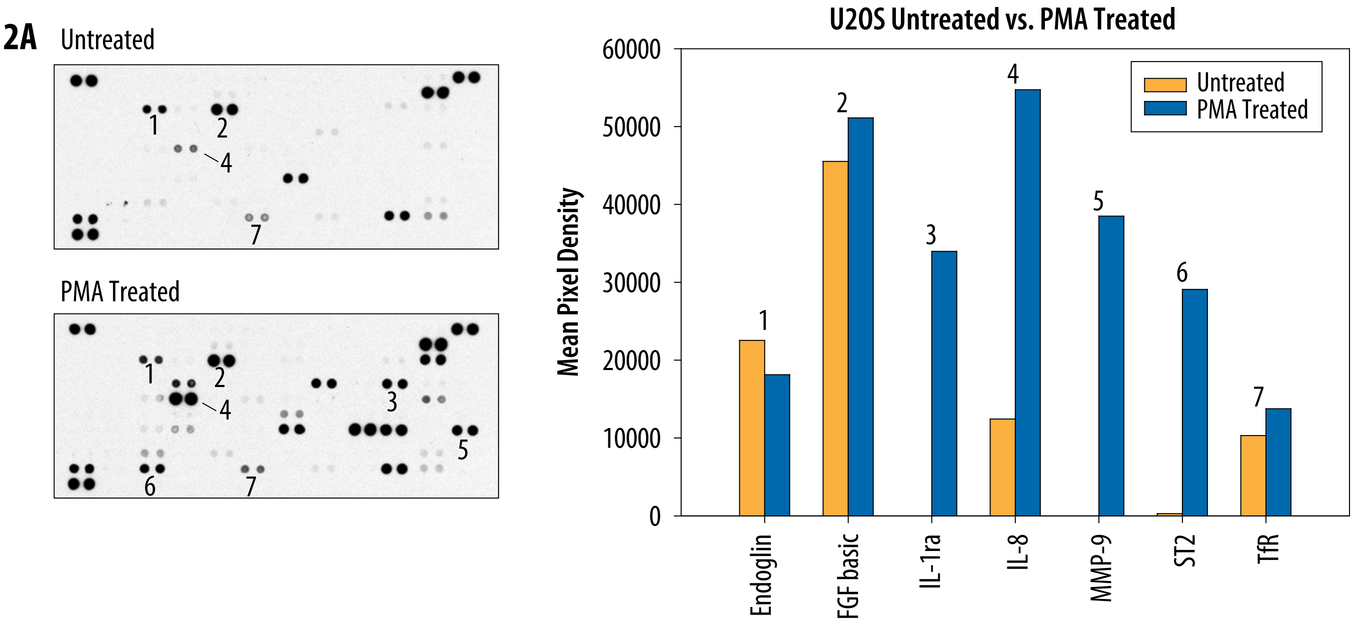

Figure 2A: U2OS human osteosarcoma cells were untreated or treated with 50 nM PMA (Tocris, Catalog # 1201) for 24 hours.

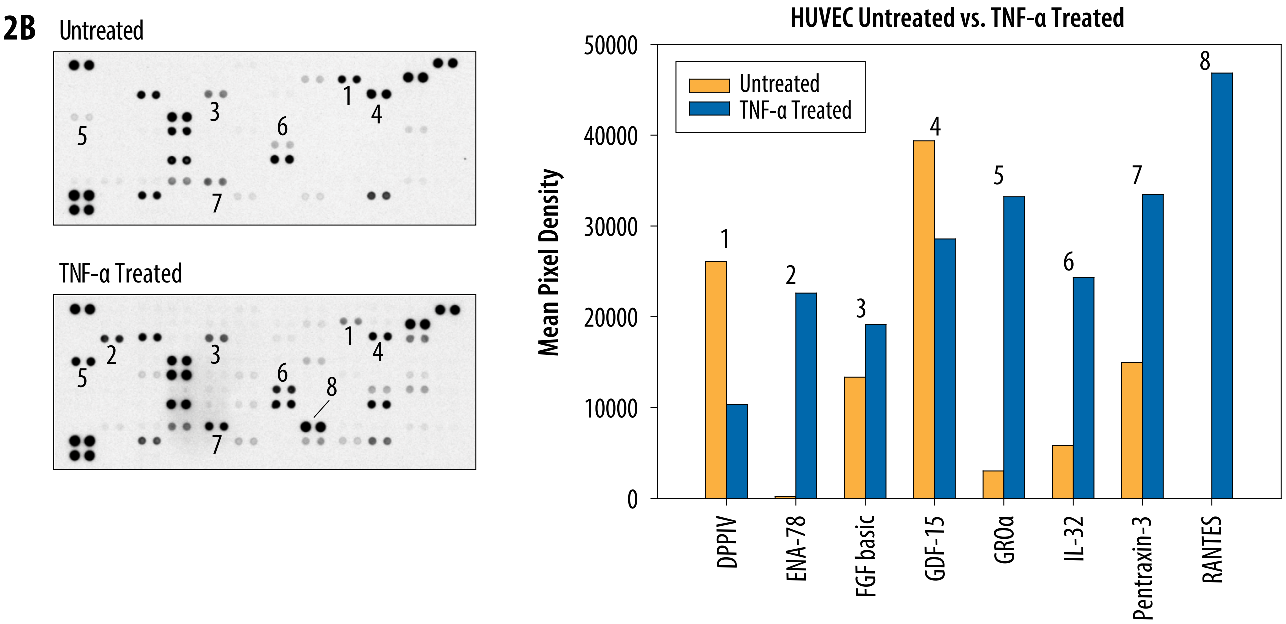

Figure 2B: HUVEC human umbilical vein endothelial cells were untreated or treated with 100 µg/mL of TNF-α (R&D Systems, Catalog # 210-TA) for 24 hours.

2C

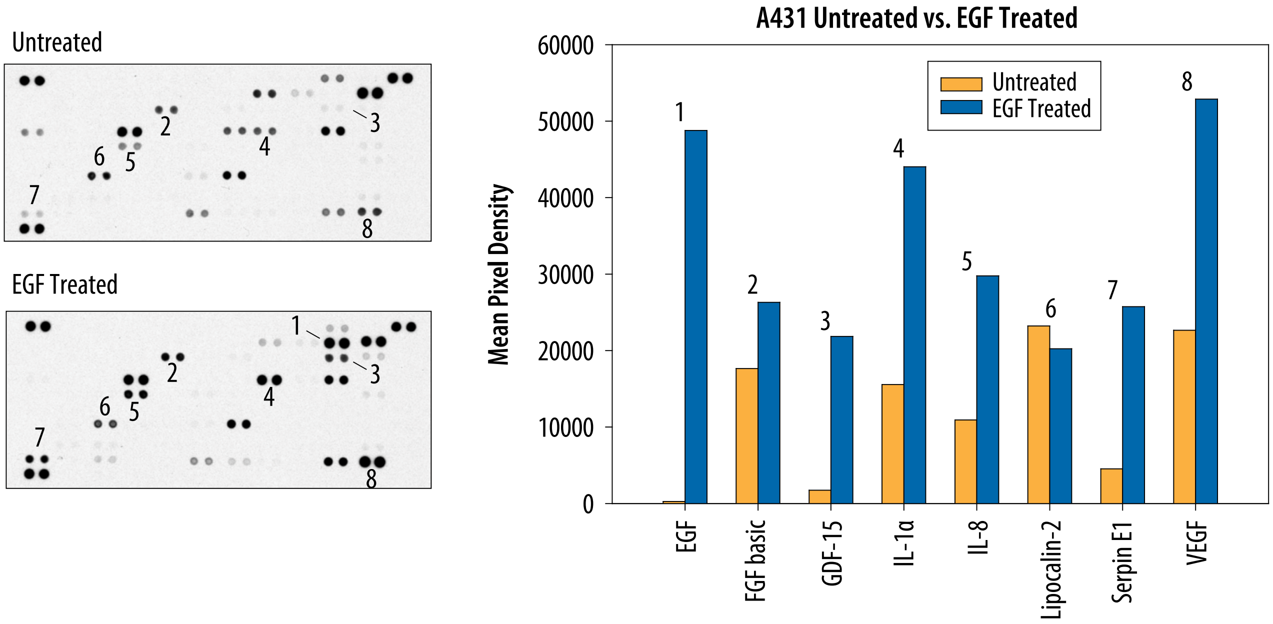

Figure 2C: A431 human epithelial carcinoma cells were either left untreated or treated with 50 ng/mL of recombinant human EGF (R&D Systems, Catalog # 236-EG) for 24 hours.

For research use only. Not for use in diagnostic procedures.

PROFILING PROTEINS IN TISSUE LYSATES

The Human XL Cytokine Array detects multiple cytokines, chemokines, growth factors and other soluble proteins in tissue lysates. 200 µg of tissue lysate was run on each array, Data shown are from a ten minute exposure to X-ray film.

Kidney

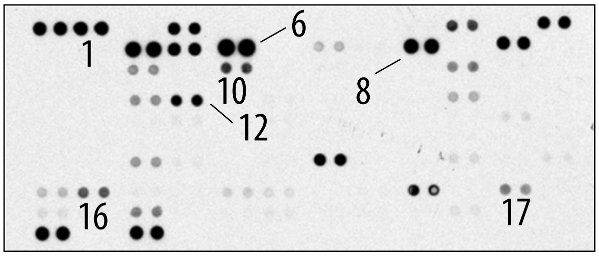

Liver

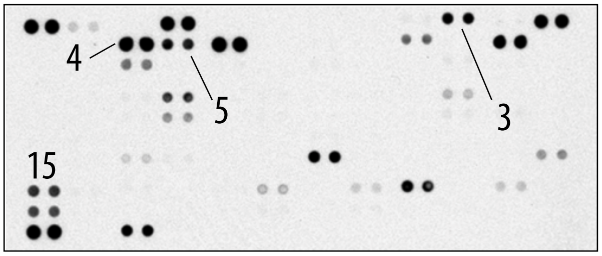

Pancreas

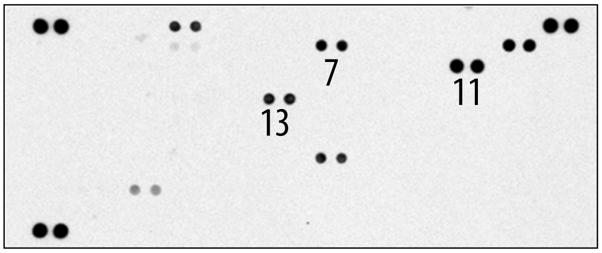

Thymus

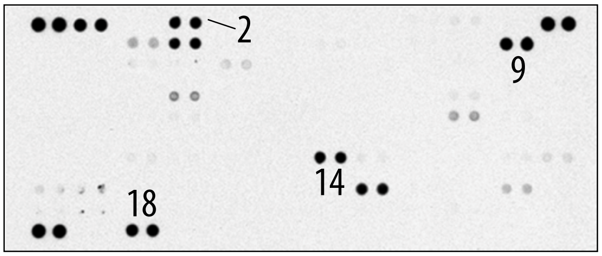

1	Adiponectin
2	Angiogenin
3	CD14
4	Chitinase 3-like 1
5	Complement Factor D
6	C-Reactive Protein
7	Cystatin C
8	DPPIV
9	EMMPRIN
10	FGF basic
11	GDF-15
12	ICAM-1
13	IGFBP-2
14	MIF
15	Myeloperoxidase
16	Osteopontin
17	Resistin
18	Vitamin D BP

PROFILING PROTEINS IN PBMC SUPERNATES & BODY FLUIDS

The Human XL Cytokine Array detects multiple cytokines, chemokines, growth factors and other soluble proteins in PBMC supernates, serum, plasma, milk, urine and saliva samples. The sample type, quantity used per array, and exposure duration to X-ray film are listed below.

A. PBMC supernates, Untreated. 500 µL per array (5 minute exposure)

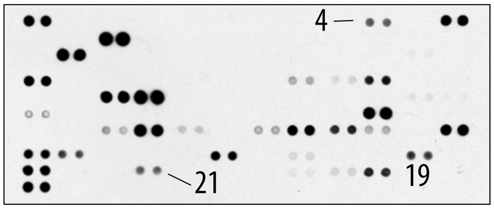

B. Serum, 200 µL per array (5 minute exposure)

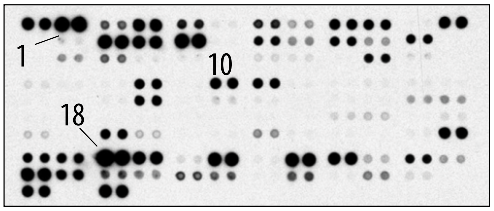

C. Citrate plasma, 200 µL per array (5 minute exposure)

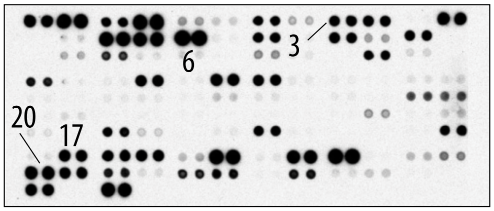

D. Human milk, 200 µL per array (5 minute exposure)

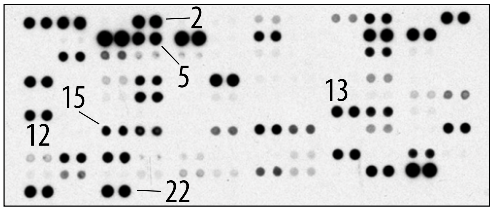

E. Urine, 200 µL per array (10 minute exposure)

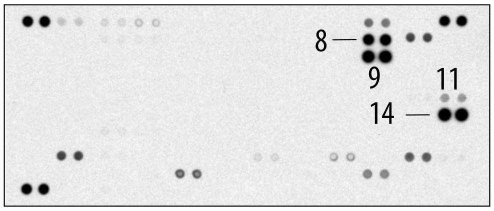

F. Saliva, 100 µL per array (10 minute exposure)

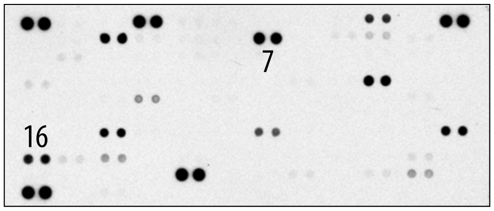

For research use only. Not for use in diagnostic procedures.

PROFILING PROTEINS IN PBMC SUPERNATES & BODY FLUIDS *CONTINUED*

		MEAN PIXEL DENSITY					
		PBMCs	Serum	Plasma	Human Milk	Urine	Saliva
1	Adiponectin	395	58,471	60,653	44,380	3006	3
2	Angiogenin	182	40,332	60,750	51,825	2683	42,927
3	Complement Component C5/C5a	0	30,460	39,246	6594	0	0
4	CD14	12,825	27,992	39,588	29,700	13,707	10,868
5	Complement Factor D	1089	43,114	58,475	49,371	407	223
6	C-Reactive Protein	16	55,506	60,897	58,931	0	0
7	Cystatin C	0	15,347	35,453	32,544	283	35,851
8	EGF	0	2953	8575	48,413	45,156	1456
9	GDF-15	0	14,413	30,389	25,317	54,828	0
10	IGFBP-2	171	32,201	48,684	48,302	75	0
11	IL-18 Bpa	408	3206	22,654	8393	8091	0
12	IL-19	5244	1057	740	35,867	65	0
13	IL-34	0	1245	187	37,624	0	0
14	Kallikrein 3/PSA	29	752	8051	0	56,276	0
15	Lipocalin-2/NGAL	7187	25,185	29,981	25,446	639	17,605
16	Myeloperoxidase	25,745	18,758	2003	2541	0	16,808
17	Osteopontin	14,627	22,065	45,824	29,169	22,878	895
18	PDGF-AA	599	60,007	41,834	34,496	295	3667
19	Resistin	16,512	13,849	6082	22,035	18,871	905
20	Serpin E1/PAI-1	33,007	49,083	53,712	707	0	216
21	TARC	13,539	2667	2574	711	0	0
22	Vitamin D BP	443	43,810	57,970	46,096	86	232

APPENDIX

Refer to the table below for the Human XL Cytokine Array coordinates.

Coordinate	Analyte/Control	Entrez Gene ID	Alternate Nomenclature
A1, A2	Reference Spots	N/A	RS
A3, A4	Adiponectin	9370	Acrp30
A5, A6	Aggrecan	176	Aggrecan 1
A7, A8	Angiogenin	283	———
A9, A10	Angiopoietin-1	284	Ang-1, ANGPT1
A11, A12	Angiopoietin-2	285	Ang-2, ANGPT2
A13, A14	BAFF	10673	BLyS, TNFSF13B
A15, A16	BDNF	627	Brain-derived Neurotrophic Factor
A17, A18	Complement Component C5/C5a	727	C5/C5a
A19, A20	CD14	929	———
A21, A22	CD30	943	TNFRSF8
A23, A24	Reference Spots	N/A	RS
B3, B4	CD40 ligand	959	CD40L, TNFSF5, CD154, TRAP
B5, B6	Chitinase 3-like 1	1116	CHI3L1, YKL-40
B7, B8	Complement Factor D	1675	Adipsin, CFD
B9, B10	C-Reactive Protein	1401	CRP
B11, B12	Cripto-1	6997	Teratocarcinoma-derived Growth Factor
B13, B14	Cystatin C	1471	CST3, ARMD11
B15, B16	Dkk-1	22943	Dickkopf-1
B17, B18	DPPIV	1803	CD26, DPP4, Dipeptidyl-peptidase IV
B19, B20	EGF	1950	Epidermal Growth Factor
B21, B22	EMMPRIN	682	CD147, Basigin
C3, C4	ENA-78	6374	CXCL5
C5, C6	Endoglin	2022	CD105, ENG
C7, C8	Fas Ligand	356	TNFSF6, CD178, CD95L
C9, C10	FGF basic	2247	FGF-2
C11, C12	FGF-7	2252	KGF
C13, C14	FGF-19	9965	———
C15, C16	Flt-3 Ligand	2323	FLT3LG
C17, C18	G-CSF	1440	CSF3
C19, C20	GDF-15	9518	MIC-1
C21, C22	GM-CSF	1437	CSF2
D1, D2	GRO-α	2919	CXCL1, MSGA-α
D3, D4	Growth Hormone	2688	GH, Somatotropin
D5, D6	HGF	3082	Scatter Factor, SF
D7, D8	ICAM-1	3383	CD54
D9, D10	IFN-γ	3458	IFNG
D11, D12	IGFBP-2	3485	———

For research use only. Not for use in diagnostic procedures.

Coordinate	Analyte/Control	Entrez Gene ID	Alternate Nomenclature
D13, D14	IGFBP-3	3486	———
D15, D16	IL-1α	3552	IL-1F1
D17, D18	IL-1β	3553	IL-1F2
D19, D20	IL-1ra	3557	IL-1F3
D21, D22	IL-2	3558	———
D23, D24	IL-3	3562	———
E1, E2	IL-4	3565	———
E3, E4	IL-5	3567	———
E5, E6	IL-6	3569	———
E7, E8	IL-8	3576	CXCL8
E9, E10	IL-10	3586	———
E11, E12	IL-11	3589	———
E13, E14	IL-12 p70	3593	———
E15, E16	IL-13	3596	———
E17, E18	IL-15	3600	———
E19, E20	IL-16	3603	———
E21, E22	IL-17A	3605	IL-17, CTLA8
E23, E24	IL-18 BPa	10068	———
F1, F2	IL-19	29949	———
F3, F4	IL-22	50616	IL-TIF
F5, F6	IL-23	51561	IL-23A, SGRF
F7, F8	IL-24	3627	C49A, FISP, MDA-7, MOB-5, ST16
F9, F10	IL-27	246778	———
F11, F12	IL-31	386653	———
F13, F14	IL-32α/β/γ	9235	———
F15, F16	IL-33	90865	C9orf26, DVS27, NF-HEV
F17, F18	IL-34	146433	C16orf77
F19, F20	IP-10	3627	CXCL10
F21, F22	I-TAC	6373	CXCL11, SCYB9B
F23, F24	Kallikrein 3	354	PSA, KLK3
G1, G2	Leptin	3952	OB
G3, G4	LIF	3976	———
G5, G6	Lipocalin-2	3934	NGAL, LCN2, Siderocalin
G7, G8	MCP-1	6347	CCL2, MCAF
G9, G10	MCP-3	6354	CCL7, MARC
G11, G12	M-CSF	1435	CSF1
G13, G14	MIF	4282	———
G15, G16	MIG	4283	CXCL9

Coordinate	Analyte/Control	Entrez Gene ID	Alternate Nomenclature
G17, G18	MIP-1α/MIP-1β	6348/6351	CCL3/CCL4
G19, G20	MIP-3α	6364	CCL20, Exodus-1, LARC
G21, G22	MIP-3β	6363	CCL19, ELC
G23, G24	MMP-9	4318	CLG4B, Gelatinase B
H1, H2	Myeloperoxidase	4353	MPO, Lactoperoxidase
H3, H4	Osteopontin	6696	OPN
H5, H6	PDGF-AA	5154	———
H7, H8	PDGF-AB/BB	5154/5155	———
H9, H10	Pentraxin-3	5806	PTX3, TSG-14
H11, H12	PF4	5196	CXCL4
H13, H14	RAGE	177	———
H15, H16	RANTES	6352	CCL5
H17, H18	RBP4	5950	———
H19, H20	Relaxin-2	6019	RLN2, RLXH2
H21, H22	Resistin	56729	ADSF, FIZZ3, RETN
H23, H24	SDF-1α	6387	CXCL12, PBSF
I1, I2	Serpin E1	5054	PAI-I, PAI-1, Nexin
I3, I4	SHBG	6462	ABP
I5, I6	ST2	9173	IL-1 R4, IL1RL1, ST2L
I7, I8	TARC	6361	CCL17
I9, I10	TFF3	7033	ITF, TFI
I11, I12	TfR	7037	CD71, TFR1, TFRC, TRFR
I13, I14	TGF-α	7039	TGFA
I15, I16	Thrombospondin-1	7057	THBS1, TSP-1
I17, I18	TNF-alpha	7124	TNFSF1A
I19, I20	uPAR	5329	PLAUR
I21, I22	VEGF	7422	BEGFA
J1, J2	Reference Spots	N/A	RS
J5, J6	Vitamin D BP	2638	VDB, DBP, VDBP
J23, J24	Negative Controls	N/A	Control (-)

All trademarks and registered trademarks are the property of their respective owners.

©2014 R&D Systems, Inc.

08.13 752859.1 6/14

For research use only. Not for use in diagnostic procedures.
